# Mapping fine-scale seagrass disturbance using bi-temporal UAV-acquired images and multivariate alteration detection

**DOI:** 10.1038/s41598-024-69695-8

**Published:** 2024-08-17

**Authors:** Jamie Simpson, Kevin P. Davies, Paul Barber, Eleanor Bruce

**Affiliations:** 1 School of Geosciences, Faculty of Science, University of Sydney, Sydney, NSW 2006 Australia; 2 Centre for CubeSats, UAVs and Their Applications (CUAVA), University of Sydney, Sydney, NSW 2006 Australia; 3 ArborCarbon Pty Ltd., Murdoch University, Rota Trans 1, Murdoch, WA 6150 Australia; 4 Centre for Terrestrial Ecosystem Science & Sustainability, Harry Butler Institute, Murdoch University, Murdoch, WA 6150 Australia

**Keywords:** Ocean sciences, Biooceanography

## Abstract

Seagrasses provide critical ecosystem services but cumulative human pressure on coastal environments has seen a global decline in their health and extent. Key processes of anthropogenic disturbance can operate at local spatio-temporal scales that are not captured by conventional satellite imaging. Seagrass management strategies to prevent longer-term loss and ensure successful restoration require effective methods for monitoring these fine-scale changes. Current seagrass monitoring methods involve resource-intensive fieldwork or recurrent image classification. This study presents an alternative method using iteratively reweighted multivariate alteration detection (IR-MAD), an unsupervised change detection technique originally developed for satellite images. We investigate the application of IR-MAD to image data acquired using an unoccupied aerial vehicle (UAV). UAV images were captured at a 14-week interval over two seagrass beds in Brisbane Water, NSW, Australia using a 10-band Micasense RedEdge-MX Dual camera system. To guide sensor selection, a further three band subsets representing simpler sensor configurations (6, 5 and 3 bands) were also analysed using eight categories of seagrass change. The ability of the IR-MAD method, and for the four different sensor configurations, to distinguish the categories of change were compared using the Jeffreys-Matusita (JM) distance measure of spectral separability. IR-MAD based on the full 10-band sensor images produced the highest separability values indicating that human disturbances (propeller scars and other seagrass damage) were distinguishable from all other change categories. IR-MAD results for the 6-band and 5-band sensors also distinguished key seagrass change features. The IR-MAD results for the simplest 3-band sensor (an RGB camera) detected change features, but change categories were not strongly separable from each other. Analysis of IR-MAD weights indicated that additional visible bands, including a coastal blue band and a second red band, improve change detection. IR-MAD is an effective method for seagrass monitoring, and this study demonstrates the potential for multispectral sensors with additional visible bands to improve seagrass change detection.

## Introduction

Monitoring of seagrass beds to detect changes and disturbances is critical for supporting conservation and restoration efforts of these important ecosystems^1–3^. Drivers of seagrass change include boat propeller scarring, natural hazards such as flood or drought, eutrophication, and climate change-induced increases in sea surface temperatures^4,5^. Disturbances to seagrass beds threaten ecosystem function and interfere with critical ecosystem services such as coastal protection, carbon sequestration, and habitat provision^6^. Direct damage to seagrass which disturbs sediments can lead to remineralisation and emission of stored carbon as CO_2_^7,8^. Conversely, successful restoration of seagrass beds can rapidly and effectively restore ecosystem function and associated ecosystem services^9,10^. Effective monitoring approaches can provide accurate information on rates of disturbance and recovery at ecologically relevant spatio-temporal scales to inform management and implementation of protection and restoration strategies^11^.

Requirements for seagrass monitoring will vary depending on the character of the ecosystem, and the nature of potential disturbances^8,12^. Consequently, approaches should consider the site and purpose. Seagrass is commonly monitored through recurrent field observations using permanent transects or quadrats, remote sensing-based methods, or a combination of these. Long-term trends in seagrass abundance or composition across a meadow can be detected using permanent quadrats and transects, which is useful for establishing the effects of large-scale change at representative sites^13,14^. Satellite remote sensing has been applied widely for measuring changes in meadow extent, especially at a regional to global scale^15–18^; however, the ability to detect finer-scale seagrass change using satellites is constrained by image spatial resolution.

Airborne-sensors combined with automatic change detection analysis techniques^19,20^ and manual image interpretation^21,22^ have been used to detect changes to seagrass at fine spatial scales. Airborne sensors can capture highly localised seagrass disturbances such as boat scars, and patch level heterogeneity in seagrass abundance which might otherwise be obscured in satellite images and permanent transect monitoring^23,24^. However, airborne data capture is relatively expensive and involves logistical challenges due to the stricter airspace and licensing requirements.

Unoccupied aerial vehicles (UAVs) provide an attractive alternative to crewed airborne data capture especially when repeat captures are required for time series monitoring. UAVs are relatively inexpensive, and the increased accessibility of UAV technology has enabled new imaging methods that can capture fine-scale features even in structurally complex meadows^25^. UAV-acquired images have been used for mapping seagrass density, ecosystem health, and species composition at fine scales^26–29^, as well as for identifying disturbances such as boat propeller scars^30^. UAVs have also supported time series seagrass monitoring with recurrent flights used to characterise seasonal change^31,32^ and identify long-term impacts of climate change on seagrass^33^.

Seagrass change detection with UAV images has used simple bi-temporal comparison of classification outputs^31,32^. This approach generally involves a supervised classification of images acquired on two different dates, and then differencing the classification outputs to determine areas of change. This relatively simple approach can be effective, but supervised classification requires suitable ground truth training and validation data for each classified image^31^, and is likely to suffer from degraded accuracy associated with post-classification change detection^34^. The requirement for extensive training and ground-truth validation data for supervised change detection methods increases time and cost burdens on coastal managers who may need to rapidly detect, investigate, and respond to potential seagrass disturbances. There is a need for a rapid, cost-effective unsupervised change detection approach to reliably map fine-scale disturbances that is not contingent on rigorous ground truth data collection.

The iteratively reweighted multivariate alteration detection (IR-MAD)^35^ method is a relatively simple, unsupervised approach for detecting bi-temporal changes in multispectral images. IR-MAD was originally developed for detecting changes in pairs of multispectral satellite images^36^ and has only had limited application to UAV images for monitoring of the built environment^37^. IR-MAD has not been applied to fine-scale monitoring of seagrass disturbances using UAV images in the literature to date.

The primary objective of this study is to demonstrate the use of the IR-MAD method for unsupervised seagrass disturbance detection using co-registered UAV images of two seagrass beds in Brisbane Water, New South Wales, Australia. Using the IR-MAD outputs, we identify key change categories and quantitatively determine the separability of change signals.

UAV sensor selection is also an important consideration for coastal managers endeavouring to use a UAV platform for coastal monitoring. There is a wide selection of commercially available sensors ranging from less expensive red–green–blue (RGB) sensors to more costly multispectral sensors with four or more bands in the visible to near-infrared range of the spectrum. The 10-band multispectral sensor used in this study (Micasense RedEdge-MX Dual) has been shown to enhance seagrass species classification^38^ and the discrimination between seagrass and macroalgae compared to sensors with only three or four bands in the visible range^39^. However, it is unclear how additional bands provided by more expensive multispectral sensors will affect the results produced by the IR-MAD method.

An additional objective of this research is therefore to assess whether sensor selection will impact on the change detection results produced by the IR-MAD method. We systematically compare IR-MAD results produced from four alternative sensor configurations. Assessing the importance of individual spectral bands for detecting seagrass change will help guide the selection of UAV sensor type when applying this method.

## Methods

### Study site

**Figure 1:**
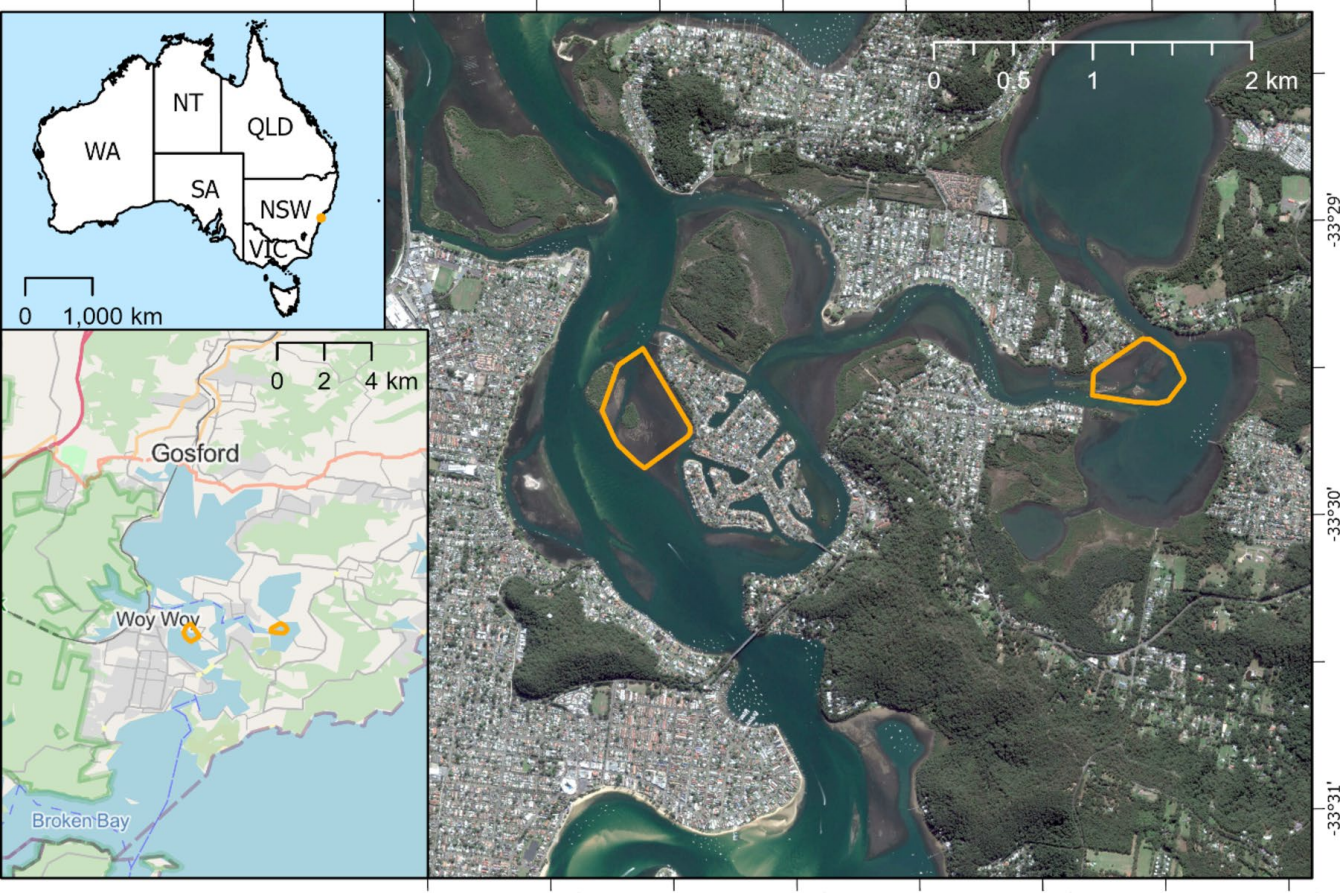
The seagrass study sites (highlighted in red) were located in the Brisbane Water estuary, NSW, Australia. The western and eastern study sites are referred to in the text as St Huberts Island and Empire Bay respectively. Map data: Google, DigitalGlobe, inset map copyright OpenStreetMap contributors and available from https://www.openstreetmap.org. Map created using ArcGIS Pro (version 3.1.0, https://www.esri.com/en-us/arcgis/products/arcgis-pro/overview).

Brisbane Water is a wave-dominated barrier estuary located ~ 45 km north of Sydney, New South Wales (NSW; Fig. 1) with a well-developed marine tidal delta and total area of ~ 27 km^2^^40^. The estuary is surrounded by residential development on all sides except Brisbane Water National Park on the western side. The area is used extensively for commercial and recreational activities, including fishing, oyster farming, boating, and swimming.

Approximately 5 km^2^ of seagrass beds are present within the Brisbane Water estuary^40^, including *Zostera capricorni* growth in shallow shoreline areas^41^, and in some areas, more extensive beds of *Z. capricorni* with *Posidonia australis* growing towards the deeper, seaward edge^42^. *Halophila ovalis* is present throughout seagrass beds in some parts of the estuary^42^.

For this study, two seagrass beds were selected, at St Huberts Island and Empire Bay, hereafter referred to by those names (Fig. 1). These beds consist primarily of *Z*. *capricorni* at varying density as well as *P. australis* which is listed as an endangered ecological community in this region of NSW^43^. The seagrass beds are in shallow depths less than 1 m at low tide, with some edges extending down to approximately 2.5 m in depth. The study sites overlap with NSW Priority Oyster Aquaculture Areas^44^ and oyster farming infrastructure is present in both seagrass beds.

### Equipment

**Figure 2:**
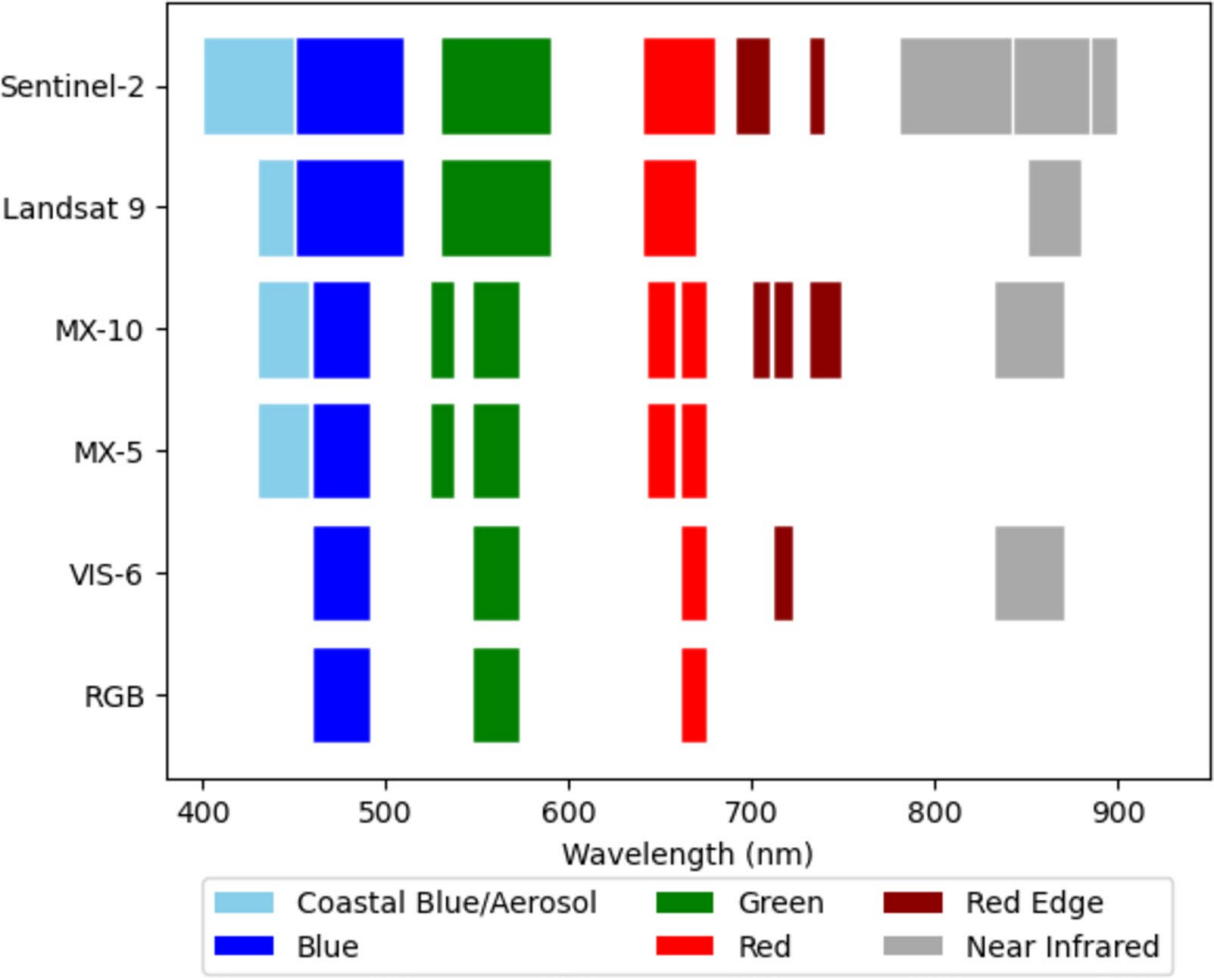
Band placements and widths for the Micasense RedEdge-MX 10 band sensor (MX-10) compared with the Sentinel-2 and Landsat 9 satellite sensors. The three alternative sensors used in this study are also shown.

Images were captured using a Micasense RedEdge-MX dual camera system (referred to as MX-10), which consists of a 5-band RedEdge-MX camera interlinked with a 5-band RedEdge-MX Blue camera providing 10 spectral bands in total (Table 1). Band subsets of each image captured by the MX-10 sensor were used to simulate three sensors with reduced spectral band coverage: the first consisted of the bands captured by the 5-band RedEdge-MX camera referred to as MX-5, and the second and third virtual sensors consisting of six and three bands referred to as VIS-6 and RGB respectively (Table 1). As the images for these three virtual sensors were created using band subsets from the original images captured by the MX-10 sensor, all images had the same spatial resolution. Compared with the band widths and placements of the Sentinel-2 and Landsat 9 satellite sensors (Fig. 2), the MX-10 sensor has a similar coastal band placement with additional bands in the green and red regions. There is also an additional band in the red-edge region compared with Sentinel-2.

**Table 1:** The sensors used in this study (MX-10) is described by the band centre and band width (FWHM).

Band	Band centre (nm)	FWHM (nm)	MX-10	MX-5	VIS-6 (virtual)	RGB (virtual)
Coastal blue	444	28	**✓**		**✓**	
Blue	475	32	**✓**	**✓**	**✓**	**✓**
Green 1	531	14	**✓**		**✓**	
Green 2	560	27	**✓**	**✓**	**✓**	**✓**
Red 1	650	16	**✓**		**✓**	
Red 2	668	14	**✓**	**✓**	**✓**	**✓**
Red Edge (RE) 1	705	10	**✓**			
RE 2	717	12	**✓**	**✓**		
RE 3	740	18	**✓**			
Near-infrared (NIR)	842	57	**✓**	**✓**

The band subsets used to represent the three virtual sensors (MX-5, VIS-6 and RGB) are also shown.

**Figure 3:**
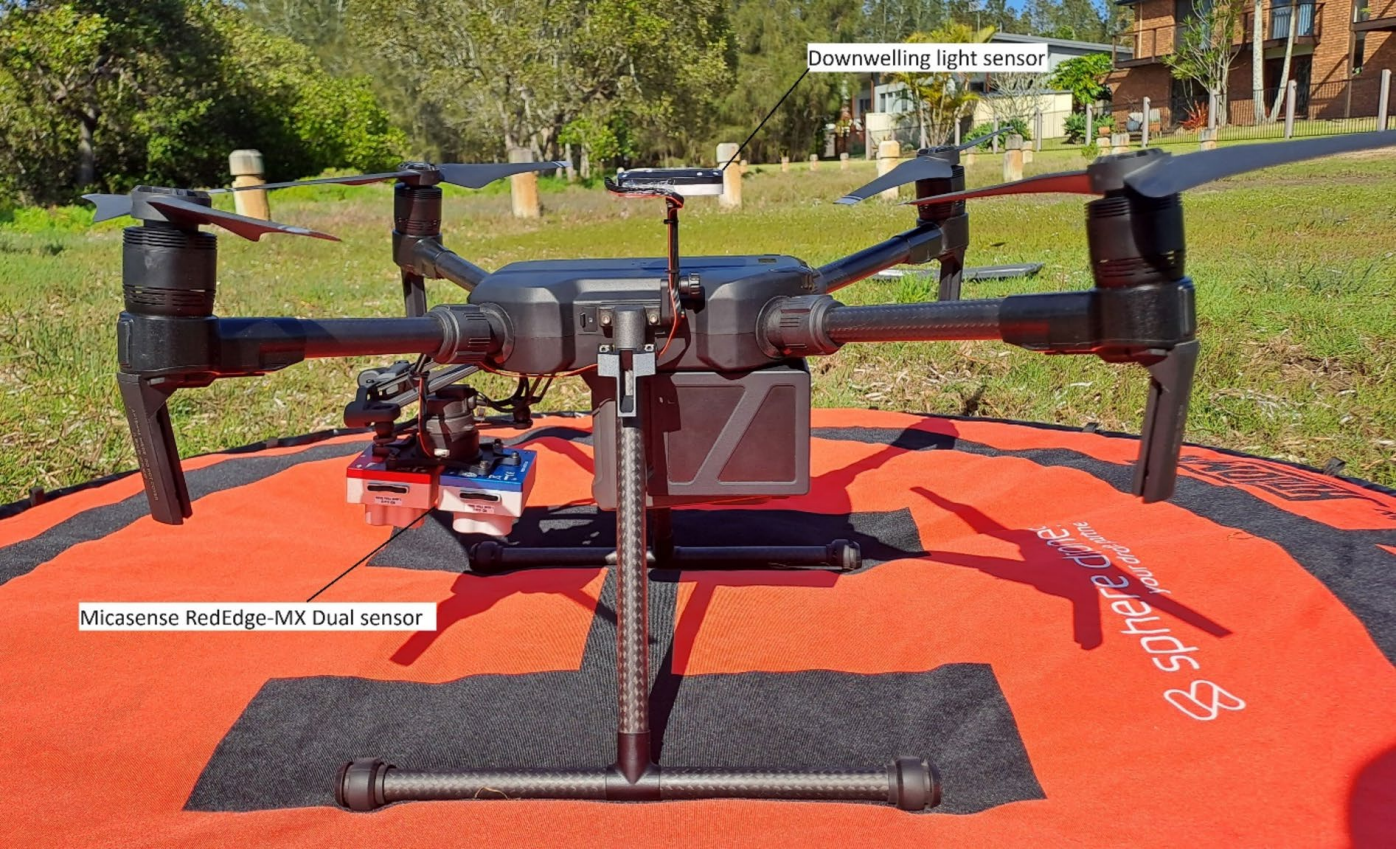
The DJI Matrice 200 UAV used in this study. The Micasense RedEdge MX Dual camera system and the downwelling light sensor are labelled. Photo by Jamie Simpson.

The camera system was mounted on a DJI Matrice 200 quadcopter UAV (DJI, Nanshan, China) with a standard Micasense RedEdge-MX Dual Camera System mount kit (Fig. 3). A downwelling light sensor was connected to the camera system and was used to measure lighting conditions at the time of capture to support radiometric calibration. The GPS contained within the camera system recorded the location of the UAV at each image capture with 3 m accuracy.

### Image capture and processing

The UAV was controlled using Measure Ground Control version 4.1.2 (AgEagle Aerial Systems Inc., Wichita, USA) in a planned “lawnmower” pattern with a 100 m flying height to achieve a spatial resolution of ~ 8 cm. On-board camera software triggered image captures based on distance travelled to achieve 75% overlap and sidelap as is considered ideal for wetland monitoring^45^. The same flight plan was used to recapture images of the study sites at an interval of 101 days (Table 2). All flights took place during below average tides with calm wind conditions (< 10 km/h), clear-sky conditions, and at low sun elevation angles to reduce potential sun glint^46^.

**Table 2:** Metadata for the UAV captures used in this study.

Study site	Image dates	Start time	End time	Tide above lowest astronomical tide	Sun elevation	Flight area	Spatial resolution
Empire bay	28/2/23	4:44pm	5:01pm	0.54m	34°	16.0 ha	8.2 cm
	9/6/23	12:03pm	12:19pm	0.50m	34°		8.0 cm
St Huberts island	28/2/23	3:19pm	3:42pm	0.46m	50°	25.3 ha	7.9 cm
	9/6/23	10:36am	11:14am	0.29m	28°		7.2 cm

The images were converted to reflectance values using images of the MicaSense Calibrated Reflectance Panel captured immediately before and after each flight, as well as data captured by the downwelling light sensor during each flight. Images were processed into multispectral orthomosaics using Metashape Professional version 2.0.2 (Agisoft LLC, St Petersburg, Russia). The first image captured for each study site was used as the reference image to georeference the second corresponding image capture. Stationary features such as large rocks, poles, and oyster farming infrastructure were used to coregister the images. As this study focused on testing a change detection method, the results were not intended to be used in conjunction with other spatial data so georeferencing to accurate real-world coordinates using real-time kinematic positioning or similar methods. Additionally, as the images vary in spatial resolution, each georeferenced set of images was spatially resampled using nearest neighbour resampling to match the coarsest resolution image for that study site (8.2 cm for Empire Bay and 7.9 cm for St Huberts Island).

### Background on IR-MAD

The goal of IR-MAD is to transform a pair of bi-temporal, multispectral images of the same scene into a set of change images with reduced dimensionality, that is, representing change in fewer bands than the original images^36,47^. IR-MAD analysis first involves performing Canonical Correlation Analysis (CCA)^48^ on a pair of geographically co-registered *N*-band multispectral images **F** and **G**. This identifies two vectors **a**_**1**_ and **b**_**1**_ which maximise the positive correlation between **a**_**1**_^**T**^**F** and** b**_**1**_^**T**^**G**, referred to as the first canonical covariate pair **U**_**1**_ and **V**_**1**_. Vectors **a**_**2**_ and **b**_**2**_ are then identified which maximise the correlation between **a**_**2**_^**T**^**F** and **b**_**2**_^**T**^**G**, with the additional constraint that the canonical variate pair **U**_**2**_ and **V**_**2**_ are uncorrelated with **U**_**1**_ and **V**_**1**_ respectively. The end result for an *N* band image pair will be N canonical variate pairs **U**_**1**_**,V**_**1**_ to **U**_**N**_**,V**_**N**_.

The canonical variate pairs are then used to produce ***N*** difference bands ***D***_***i***_** = *U***_***i***_***–V***_***i***_, referred to as the MAD variates. The MAD variate pixel values are expected to be normally or near-normally distributed, and pixels with more positive or negative values represent greater change. As the canonical variate pairs were ordered by decreasing correlation, the MAD variates will be ordered from least to most change information, referred to as lowest to highest order. Given the canonical variate pairs were uncorrelated with each other, each MAD variate is expected to contain different types of change.

The sum of the MAD variates is chi-squared distributed, and no-change pixels are identified using a pre-determined threshold of the probability density function of this distribution. The MAD process is then repeated with no-change pixels receiving a higher weight during the CCA. This iteration is performed until the correlation between the first canonical variate pair is maximised. The iterative reweighting of no-change pixels results in an improved transformation which better represents change in the final MAD variates^35^. The three MAD variates containing the most change information can then be displayed in false colour to visualise change that incorporates information from all the original bands but with reduced dimensionality^47^.

An important quality of the IR-MAD method for unsupervised change detection is that IR-MAD is insensitive to linear scaling effects between the original image pairs (such as due to differences in atmosphere, illumination, calibration, and sensor response) because the CCA finds linear combinations of the original spectral bands^49^.

### Application of IR-MAD to the alternative sensors

To assess the relative importance of sensor selection, and the importance of additional spectral bands for detecting change to seagrass, four alternative sensor configurations (Table 1 and Fig. 2) were compared in the IR-MAD analysis below. These were the dual MX-10 sensor, the single MX-5 sensor, as well two virtual sensors constructed from band subsets captured by Micasense sensors. The first virtual sensor represented a conventional RGB camera, and images were constructed from the standard red, green, and blue bands captured by the MX-5 sensor. The second virtual sensor represented a theoretical sensor with additional visible bands to a conventional 3-band RGB sensor. Images for this sensor were constructed from the six shortest-wavelength visible light bands from the MX-10 sensor (referred to as the VIS-6 sensor). The red-edge and near-infrared bands from the MX-10 sensor were not included in the VIS-6 sensor because they are known to have poor water penetration^50^.

The IR-MAD method was applied to the bi-temporal image pair captured by the MX-10 sensor for each study site using the The *CRCPython* library^49^. The IR-MAD method was then then repeated for the corresponding image pairs for the MX-5, RGB, and VIS-6 sensors for each study site. The result was four IR-MAD outputs for each study site.

### Qualitative identification of change categories

To examine the effectiveness of IR-MAD for detecting fine-scale changes to seagrass, the output MAD variates for the MX-10 sensor for each study site were visually assessed to identify and categorise different types of change. MAD variates were first viewed individually to identify the number of variates that contain spatially coherent change features. Notable change signals were then identified and inspected in the original bi-temporal image pairs to qualitatively determine the type of seagrass change.

Six different categories of change were visually identified (Table 3), and their boundaries were manually digitised using the Region of Interest (ROI) Tool in ENVI version 5.6 (NV5 Geospatial Solutions, Broomfield, United States). Separate ROIs were also created for two types of unchanged areas with seagrass present in both images: one without any change signal in the IR-MAD data (Unchanged 1) and one with a false positive change signal (Unchanged 2).

**Table 3:** Identified change categories and descriptions of change signals identified in IR-MAD outputs, and whether the signal is a false positive indication of change.

Change category	Description of change	Actual change?
Propeller scar	Seagrass changed to bare ground in long, linear features, caused by damage from boat propellers	Yes
Damaged patch	Seagrass changed to bare ground in larger patches, caused by damage from sources other than propellers, such as boat groundings or seagrass die-off	Yes
Regrowth	Bare ground changed to seagrass, possibly caused by seagrass regrowth	Yes
Added oyster cage	Oyster infrastructure added over seagrass bed	Yes
Removed oyster cage	Oyster infrastructure removed from seagrass bed	Yes
Bright sand false positive	Apparent change signals recorded over unchanged sand patches	No
Unchanged 1	Unchanged seagrass or bare ground, submerged in both images	No
Unchanged 2	Unchanged seagrass or bare ground, submerged in one image and above water in the other	No

### Comparing effectiveness of disturbance detection between the alternative sensors

In order to quantitatively assess the ability to distinguish the different types of identified change types in the IR-MAD outputs for each of the alternative sensors, the spectral separability between the different change categories was quantitively determined using the Jeffreys-Matusita (JM) distance^51^. The JM distance is a measure of separability between two sets of probability distributions, commonly used in remote sensing to determine spectral separability of class endmembers to support supervised classifications. The JM distance $J$ between two distributions $x$ and $y$ is defined as^52^:

1 $$\begin{array}{c}{J}_{xy}=2\left(1-{e}^{-B}\right),\end{array}$$

where

2 $$\begin{array}{c}B= \frac{1}{8} {\left({\mu }_{x}-{\mu }_{y}\right)}^{T}{\left(\frac{{\sum }_{x}+{\sum }_{y}}{2}\right)}^{-1}\left({\mu }_{x}-{\mu }_{y}\right)+ \frac{1}{2}\text{ln}\left(\frac{\left|\frac{{\sum }_{x}+{\sum }_{y}}{2}\right|}{{\left|{\sum }_{x}\right|}^\frac{1}{2}{\left|{\sum }_{y}\right|}^\frac{1}{2}}\right),\end{array}$$

where ${\mu }_{x}$ and ${\mu }_{y}$ are the mean vectors of $x$ and $y$, and ${\sum }_{x}$ and ${\sum }_{y}$ are the covariance matrices of $x$ and $y$.

JM distance values can range from 0 (no separability) to 2 (perfect separability). JM distances can therefore be compared between class pairs in remote sensing images as a measure of statistical separability of different features. For IR-MAD variates, JM distance values can similarly be used as a measure of relative statistical separability of different features (in this case change features) in the image.

The JM distance was used to determine whether the ROIs captured for areas representing actual change were separable from ROIs where no-change occurred, and if ROIs representing different change categories could be distinguished from each other. The JM distance was calculated for each pairwise combination of the eight change ROIs for the MX-10 sensor. This was then repeated for the other three sensors (MX-5, RGB, and VIS-6) to compare the relative strength of detecting change between the four sensors.

### Assessing individual band influence on change detection using IR-MAD

One of the non-image outputs from the IR-MAD analysis is the set of eigenvector pairs (**a**_**1..N**_ and **b**_**1..N**_**).** The eigenvectors contain the final weights applied to the original spectral bands used to produce the canonical variate pairs and the derived MAD variates. The eigenvectors were therefore used to explore which spectral bands in the original bi-temporal image pair had the most influence on the generation of the MAD variate bands. This provides further information to support sensor selection by indicating which bands are most important for change detection.

Vectors **a**_**i**_ and **b**_**i**_ are of length equal to the number of input bands to the IR-MAD process. Therefore, for the bi-temporal image pair captured by the MX-10 sensor at St Huberts Island, the 1^st^ elements of the eigenvectors **a**_**1**_ and **b**_**1**_ represents the weights applied to the pair of coastal blue bands used (in part) to produce the first canonical variate pair (**CV**_**1**_ and **CV**_**2**_) and subsequent MAD variate. Relatively higher weights in vectors **a**_**i**_ or **b**_**i**_ indicate a greater degree of influence by the corresponding band on the resulting canonical variate pair, and corresponding MAD variate.

The vector weights produced by the IR-MAD method for different image pairs are not directly comparable. To compare the influence of individual bands across different IR-MAD analysis the eigenvector elements were transformed based on their relative contribution to the CCA transformation, and the number of bands present in the image pair. Elements from each eigenvector were selected corresponding to the MAD variates in which spatially coherent change features were present. The absolute values of these elements were averaged and normalised to determine the relative contribution of each band, and each weight scaled to the full number of bands in the original bi-temporal image pair:

3 $$\begin{array}{c}{W}_{n}= {n}_{max} \times \frac{1}{N}{\sum }_{i=1}^{N}\frac{{|a}_{i}\left(n\right)\left|+{|b}_{i}\left(n\right)\right|}{2},\end{array}$$

where ${a}_{i}(n)$ and ${b}_{i}(n)$ are the pair of eigenvector elements for band $n$, $N$ is the number of IR-MAD bands which contain spatially coherent elements, ${W}_{n}$ is the scaled transformed weight for the spectral band $n$ corresponding with the eigenvector element $n$, and ${n}_{max}$ is the number of bands in each image in the bi-temporal image pair.

The higher the scaled transformed weights for a band in the eigenvector corresponding with a MAD variate, the more those bands influenced the derivation of that MAD variate.

The normalised and scaled eigenvector weights (${W}_{n}$) was used to compare the relative contribution of each spectral band for detecting changing using IR-MAD from images captured by the four alternative sensors used in this study.

## Results

### IR-MAD analysis for the alternative sensors

**Figure 4:**
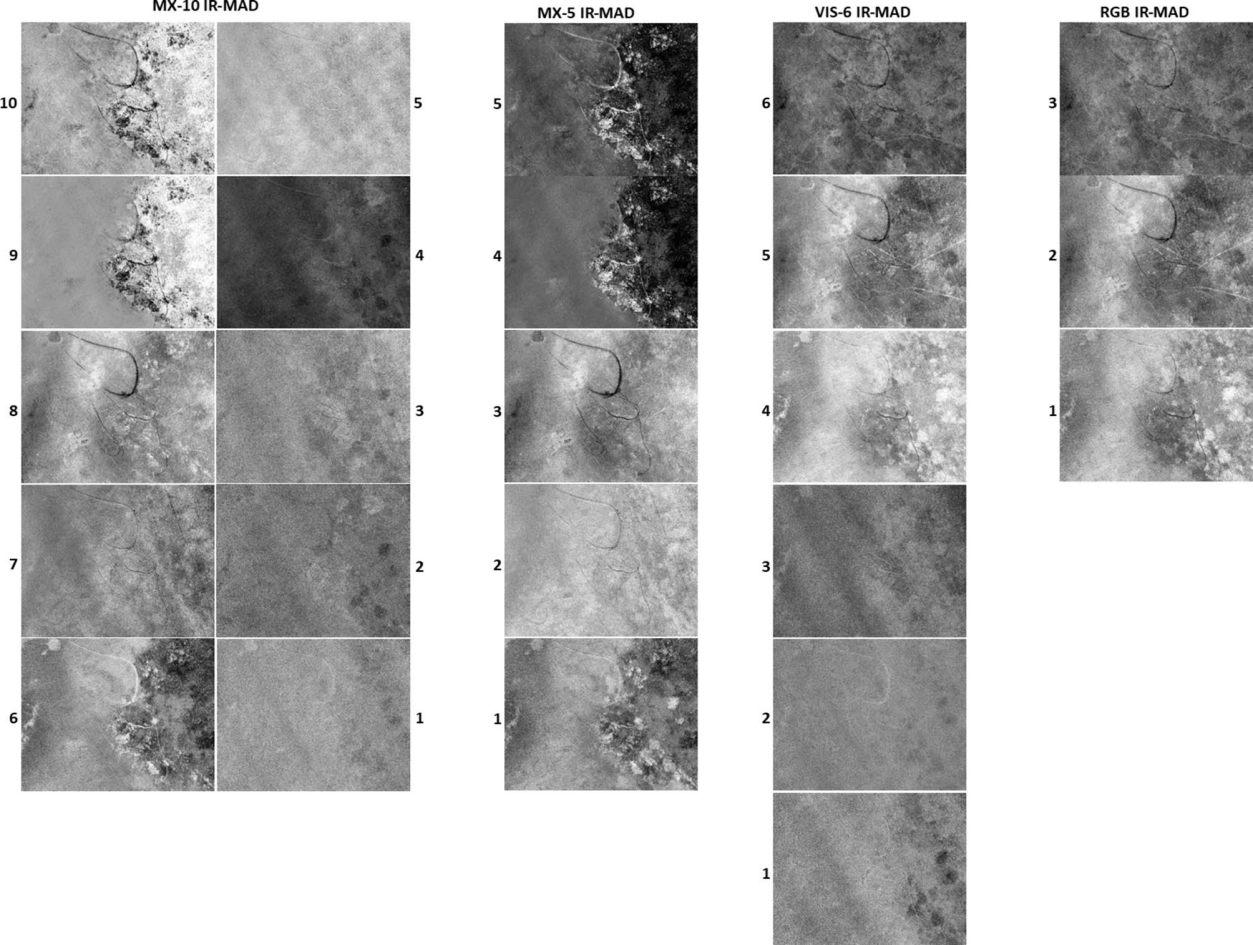
MAD variates for each of the four sensors for a subset of the St Huberts Island study site. Each MAD variate is visualised with a 5% linear greyscale stretch. MAD variates are numbered from 1 representing the lowest order.

For each study site, four sets of MAD variates were produced for the four alternative sensors. Spatially coherent change was visually present in the higher-order MAD variates, while the lower-order variates consisted primarily of noise (Fig. 4). Spatially coherent change features were visible in the five highest order variates (6 to 10) derived from the MX-10 sensors, three variates (4 to 6) derived from the VIS-6 sensor, and for all the variates derived from both the MX-5 and RGB sensors.

### Qualitative identification of change categories

**Figure 5:**
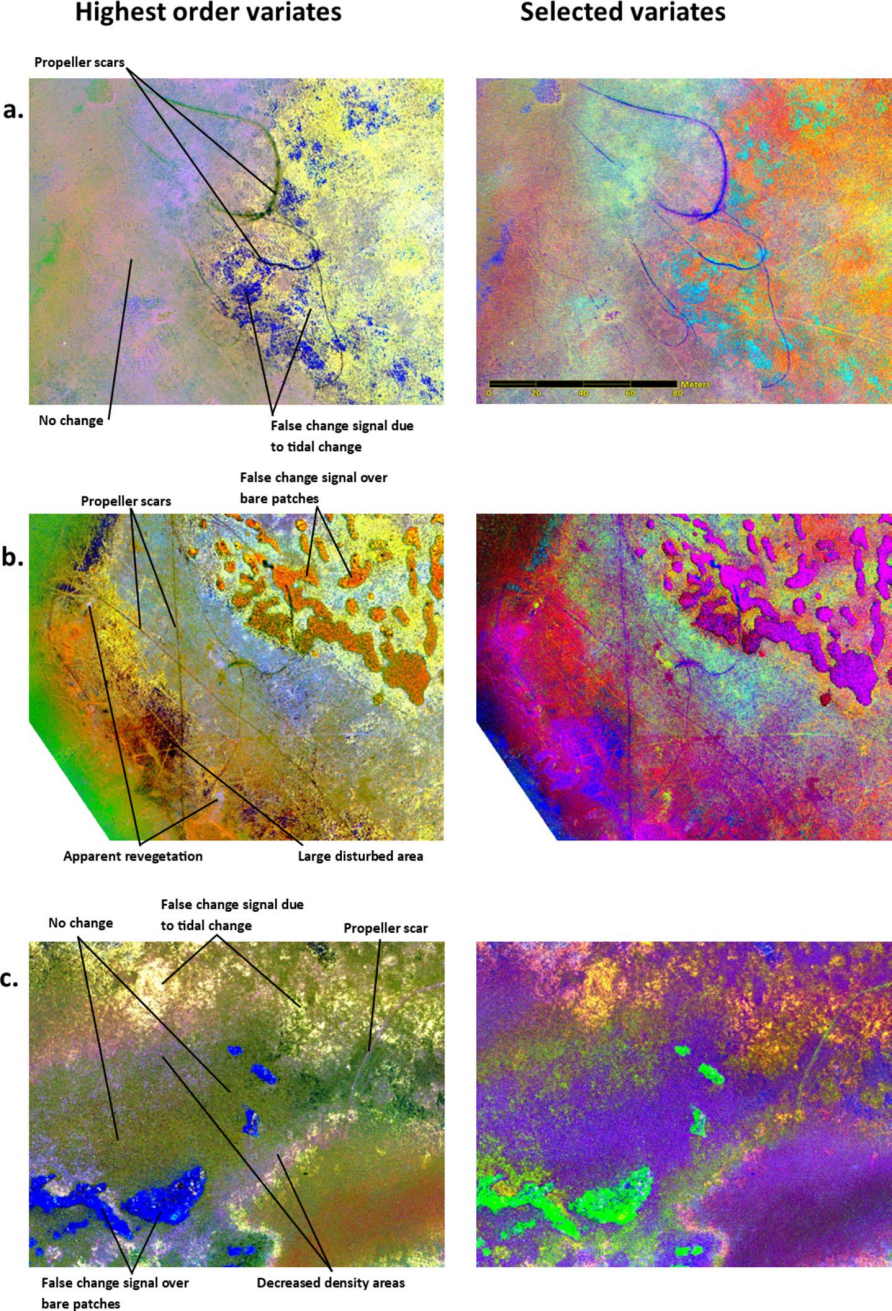
Examples of identified change categories overlayed on false colour change images. Change images were derived using the higher-order MAD variates 10, 9, and 8 (left column), variates 10, 8, and 6 (right column, image (**a**) and (**b**)), and variates 9, 8, and 7 (right column, image (**c**)). Change categories include: (**a**) propeller scars at St Huberts Island, (**b**) a large disturbed area at St Huberts Island, and (**c**) areas of decreased seagrass density at Empire Bay. All variates were derived from bi-temporal images captured by the MX-10 sensor.

Spatially coherent changes were visible in the false colour IR-MAD output images for the MX-10 Sensor (annotated in Fig. 5). These changes are visualised with two different sets of variates: the highest order MAD variates in the left column (variates 10, 9, and 8) and variates manually selected to highlight disturbances in the right column (variates 10, 8, and 6 for St Huberts Island; variates 9, 8, and 7 for Empire Bay). These change categories included boat propeller scars, a large area of disturbance, seagrass patches which decreased in density without clear evidence of direct disturbance, apparent regrowth of seagrass, and false positive change signals.

Linear propeller scars and non-linear disturbed patches, likely anthropogenic, are visible across the St Huberts Island study site. At the Empire Bay study site, fewer disturbances are visible, including a single propeller scar and decrease in cover density at the edge of the seagrass bed. Existing propeller scars present in both images result in IR-MAD values indicating no change.

New propeller scars and a larger disturbed area are clearly visible in all three locations shown in Fig. 5. In the false colour images of St Huberts Island, these features appear black-green when visualised with the highest-order variates (left column) and blue when visualised with selected variates (right column) (Fig. 5). In the images from Empire Bay, propeller scars are light purple and green in the false colour images created using highest-order variates and selected variates respectively (Fig. 5). Other parts of Fig. 5 show general decreases in seagrass density across patches, which show a distinct spatial pattern to the boat damage in the St Huberts Island locations. These decreases in seagrass density may be related to disturbance, or to seasonal variation in seagrass density.

**Figure 6:**
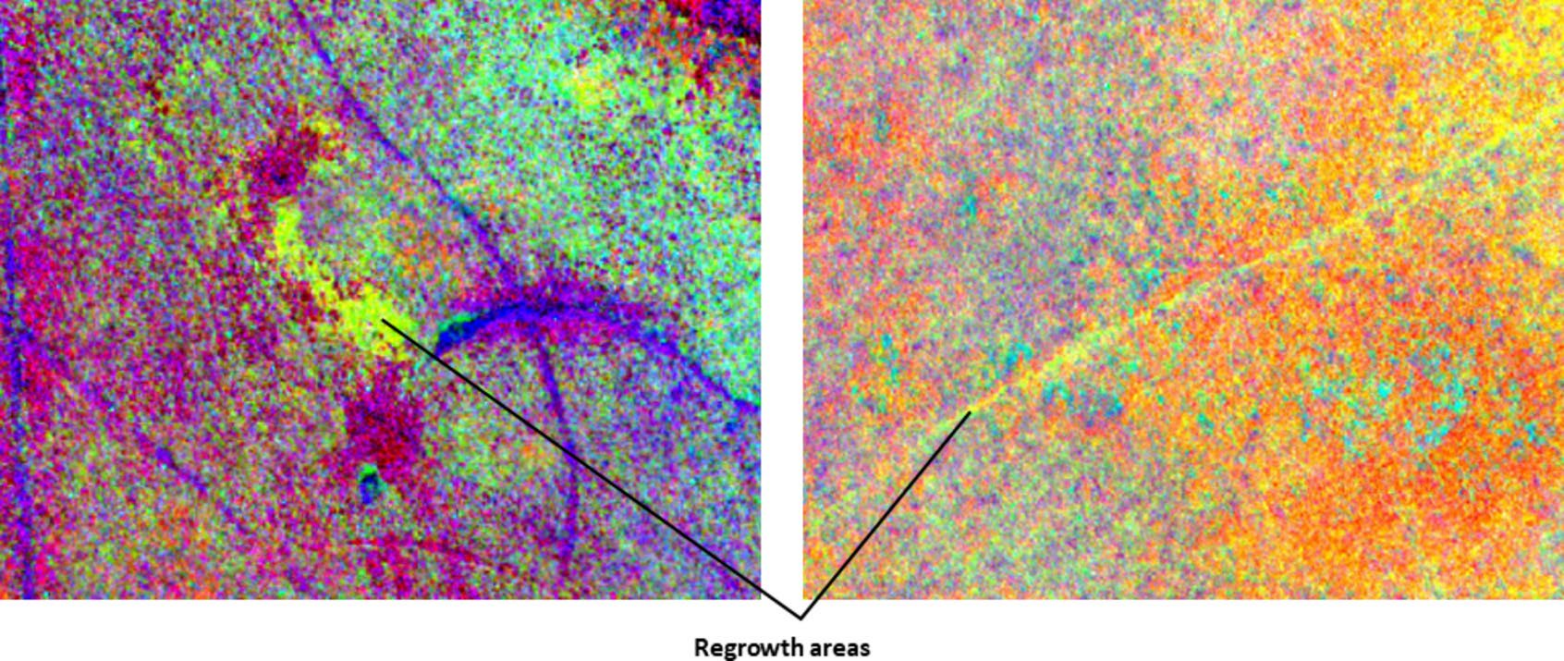
Apparent seagrass regrowth annotated on spatial subsets of false colour images of the IR-MAD outputs (variates 10, 8, and 6) for St Huberts Island (derived from the MX-10 sensor).

Apparent regrowth of seagrass was also identified in the St Huberts Island study site (Fig. 6). These areas appear as bare ground in the February image and vegetation in the June image. This pattern may indicate (1) seagrass regrowth over previously bare sediment or propeller scars; (2) seagrass leaves changing in position due to water flow, or (3) spatial inaccuracies in image registration or orthorectification.

**Figure 7:**
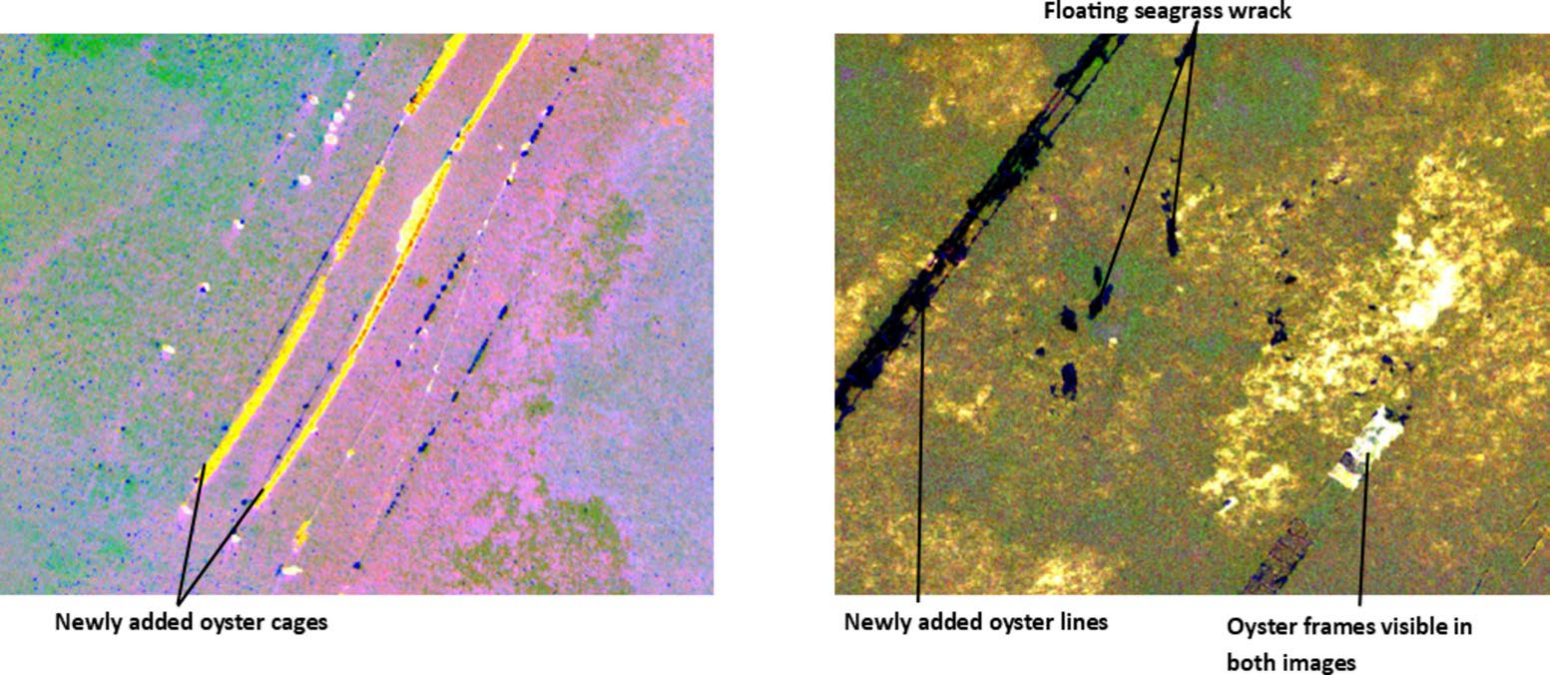
Oyster aquaculture infrastructure changes identified in the IR-MAD outputs for St Huberts Island (left) and Empire Bay (right). Both images were visualised using MAD variates 10, 9, and 8 derived from the bi-temporal image pairs captured by the MX-10 sensor.

The other change categories identified in the qualitative analysis of the St Huberts Island study site included changes related to oyster farms. The oyster farms consist of cages or trays that can be attached to lines as needed and are frequently moved depending on changes to water conditions. These operational practices were identified in the IR-MAD outputs for both study sites (Fig. 7).

### Comparing effectiveness of disturbance detection between the alternative sensors

The JM distance values for each class pair are a relative measure of statistical separability, indicating how effectively the change category can be distinguished from others. The JM distance values were calculated across all change category pairs for all four alternative sensors (Table 4). For ease of comparison between sensors in the text, the JM distance results were arbitrarily grouped into three classes–Strong (JM distance ≥ 1.8), Moderate (1.8 > JM distance ≥ 1.4), and Weak (1.4 > JM distance) (Table 5).

**Table 4:**
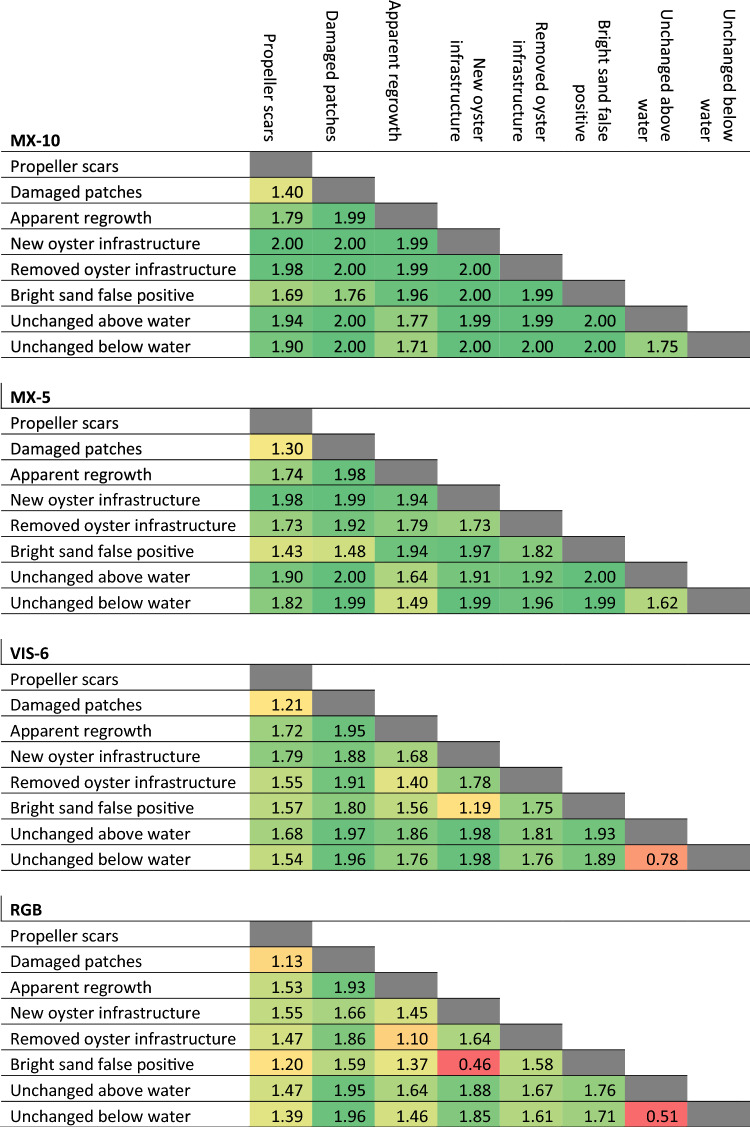
JM distance values between the eight identified change categories for the four sensors: MX-10, MX-5, VIS-6, and RGB.

Table cells colour coded from weakest separability (red) to strongest (dark green).

**Table 5:** Number of change category pairs with strong (JM distance > 1.8), moderate (1.8 ≥ JM distance > 1.4), and weak (1.4 ≥ JM distance) separability for each alternative sensor.

Sensor	Strong	Moderate	Weak
MX-10	21	6	1
MX-5	18	6	4
VIS-6	12	12	4
RGB	6	11	11

Based on JM distance calculated from each alternative sensor's IR-MAD results.

The IR-MAD outputs for the MX-10 sensor had the highest JM distance values (Table 4), with 21 change category pairs showing strong separability (Table 5). Propeller scars and damaged patches had relatively low separability from each other (JM distance 1.40) but moderate to strong separability from all other classes. Apparent regrowth and changes to oyster infrastructure also showed moderate to strong separability from all other classes.

A total of 18 change category pairs had strong separability for the IR-MAD variates derived from the MX-5 sensor (Table 5). Like the MX-10 data, the lowest separability was between the propeller scars and damaged patches (Table 4). These disturbances were only moderately separable from the bright sand false positive class in this dataset but were relatively more separable from the others.

JM distances for the VIS-6 sensor (Table 4) showed 12 strongly separable class pairs (Table 5). Disturbed patches and propeller scars had strong (JM distance ≥ 1.80) and moderate (JM distance ≥ 1.54) separability respectively from all classes except each other (JM distance 1.21). Apparent regrowth showed moderate to strong separability from all other classes except removed oyster infrastructure.

The JM distance results derived from the RGB sensor (Table 4) showed the weakest separability values compared with other sensors. Larger disturbed patches displayed strong or moderate separability from all classes except propeller scars. However, propeller scars showed weak separability (JM distance < 1.5) from most other classes, including bright sand false positives and both unchanged classes. Apparent regrowth was separable from both disturbed classes, but showed weaker separability from changes to oyster infrastructure, and the below water unchanged class.

**Figure 8:**
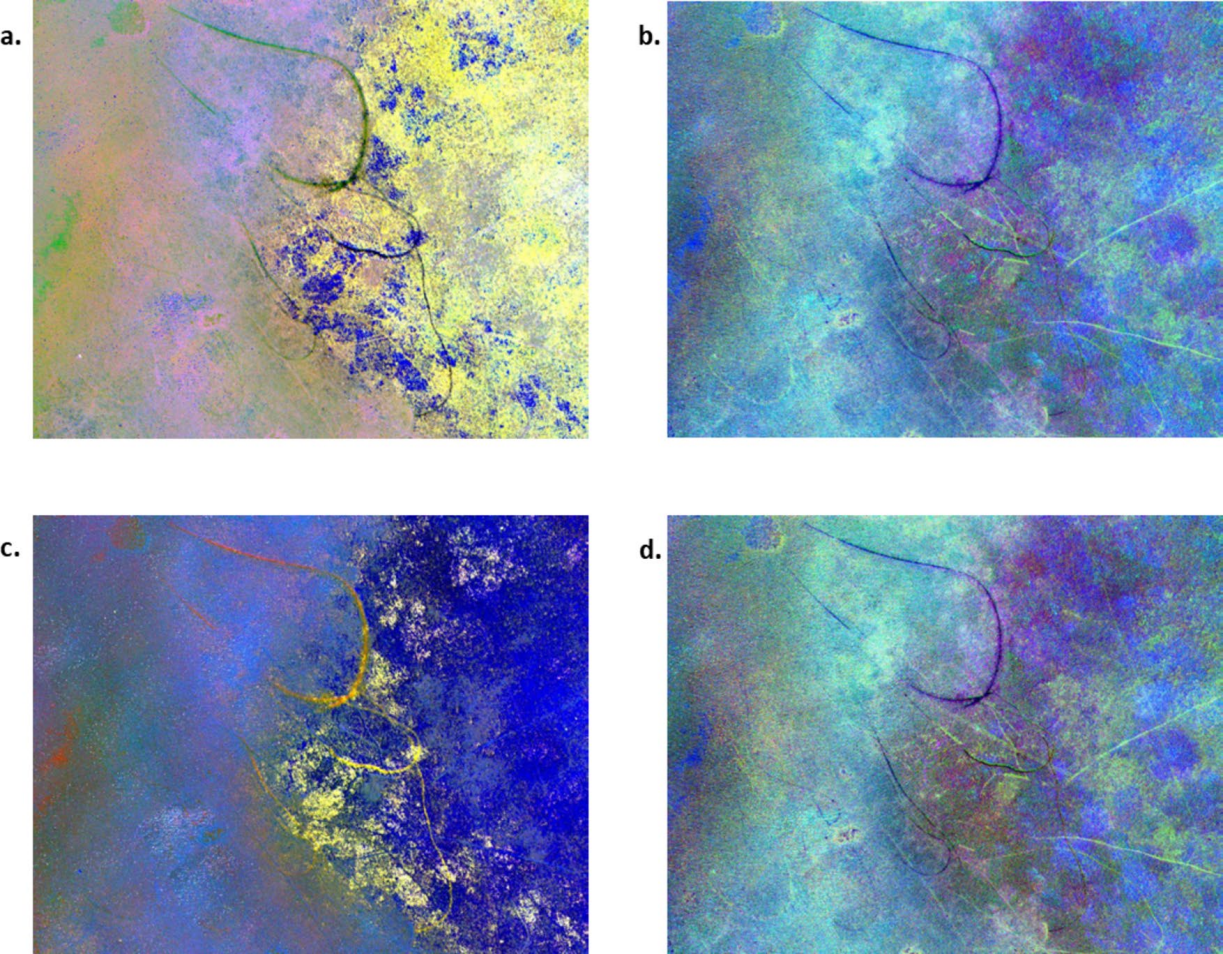
Propeller scars were visibly present at the St Huberts Island in the (**a**) MX-10 sensor IR-MAD outputs visualised with variates 10, 9, and 8, (**b**) VIS-6 sensor IR-MAD output visualised with variates 6, 5, and 4, (**c**) MX-5 sensor IR-MAD output visualised with variates 5, 4, and 3, and (**d**) the RGB sensor IR-MAD output visualised with variates 3, 2, and 1.

Separability between the two unchanged classes was lower for the two virtual (VIS-6 and RGB) sensors which did not have the red-edge and near-infrared. Submerged unchanged areas and emerged unchanged areas appear more similar in the VIS-6 and RGB sensor IR-MAD outputs data compared to the MX-10 and MX-5 IR-MAD outputs (Fig. 8).

### Individual band influence on detecting change

The transformed eigenvector weights were used to assess the contribution of each band to the IR-MAD calculations across both study sites for all four alternative sensors (Table 6).

**Table 6:**
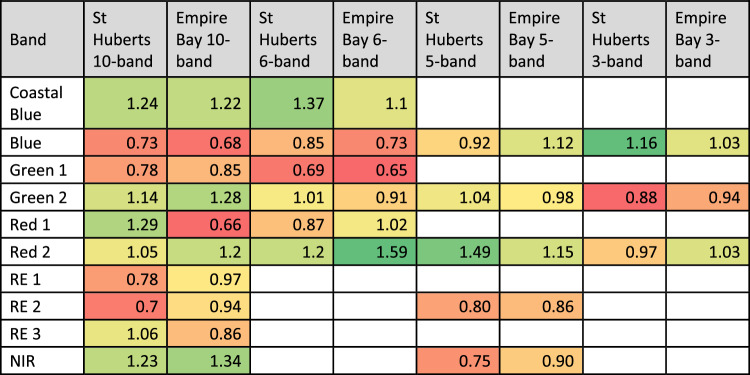
Transformed weights for each eigenvector element, indicating the influence of each corresponding spectral band on MAD outputs.

Across the data from the MX-10, VIS-6, and MX-5 sensors, the Green 2 and Red 2 bands had a consistently high influence, while the Blue band was consistently less influential on the IR-MAD results. For the RGB sensor data, the blue band had the highest influence. The NIR band in the MX-10 sensor data also had a relatively large statistical influence on the change signals produced by this sensor. The five MX-10 sensor bands that were not available in the MX-5 sensor data had differing influences on change detection results. The Green 1 band had a relatively small influence, the Red 1 band had a variable influence depending on the image pair, and the Coastal Blue band had a consistently high influence.

## Discussion

The IR-MAD method applied to images captured by a UAV was shown to be viable for detecting fine-scale physical disturbances to seagrass beds using the 10-band, 5-band, and 6-band data, and potentially effective for 3-band data. Large areas of disturbed seagrass were visibly identified in IR-MAD results from all sensors and were strongly separable from all other change categories in the 10-band and 5-band data, and from most other change categories in the 6-band and 3-band data. Propeller scars were visible in all IR-MAD outputs, and JM distance results showed moderate to strong separability for propeller scars in the 10- and 5-band data, moderate separability in the 6-band data, and weak to moderate separability in the 3-band data. This study demonstrates the potential of IR-MAD analysis to support seagrass monitoring and management at relevant finer spatio-temporal resolution, especially when multispectral sensors like the Micasense RedEdge-MX Dual sensor are used.

Substantial changes in seagrass cover were identified at both study sites, but they were noticeably different in the IR-MAD results. At St Huberts Island, many propeller scars and other instances of boat damage were identified, clustered towards the western edge of the meadow and near oyster aquaculture infrastructure, where boat traffic is likely to be highest. At Empire Bay, however, the patterns of change were more spatially consistent potentially indicating seasonal trends or environmental conditions such as water quality.

The full set of 10 bands provided by the MX-10 sensor demonstrated advantages for seagrass change detection. The IR-MAD method using images from the MX-10 sensor detected change features more effectively than the other three sensors, with strongly separable JM distance measurements recorded for all eight change categories tested. Outputs from the theoretical VIS-6 sensor also offered improved change detection over the RGB sensor. Two of the additional visible bands included in IR-MAD analysis for the MX-10 and VIS-6 sensors (Coastal Blue and Red 1), had a relatively high weighting, indicating a strong influence on change detection. This suggests that their addition enhances change detection compared to the MX-5 sensor.

The improvement offered by these additional bands is consistent with past research using satellite-borne sensors for characterising seagrass in coastal ecosystems. For example, the Sentinel-2 coastal blue band was shown to improve seagrass classification^53^, and analysis of hyperspectral images has shown that the green–red visible range is optimal for predicting seagrass percent cover and related variables^54–56^. Multispectral sensors with additional visible light bands compared with a standard RGB sensors has been demonstrated here and elsewhere^57^ to provide improved ability to characterise seagrass ecosystems without necessitating the use of expensive hyperspectral sensors.

Though the full 10 band MX-10 sensor was the most effective in discriminating change classes, the change class separability results suggest that cheaper and simpler sensors may be sufficient for efficient monitoring of seagrass using IR-MAD. The Micasense RedEdge MX sensor (MX-5) is a commonly available sensor which can be integrated on consumer UAVs at relatively low cost. For visual analysis of IR-MAD outputs, the 5-band images provided by this sensor has been demonstrated here as sufficient for focused management responses to seagrass disturbance.

The red-edge and near-infrared bands can improve accuracy results in seagrass mapping when leaves are on or near the surface of the water due to the absorption of red and scattering of near-infrared light by photosynthetic vegetation^26^. However, the high absorbance of red-edge and near-infrared light by water can reduce the effectiveness of bands in these regions when seagrass is fully submerged. The IR-MAD analyses in this study which included red-edge and near-infrared bands detected differences in water level between image captures as a distinct category of change which is separable from disturbance events, and this was reflected in the high level of influence that the NIR band had on the MX-10 change result. However, IR-MAD outputs produced without the red-edge and near-infrared bands are easier to interpret visually as the extensive change signals caused by tidal differences are less apparent, highlighting the change signals representing actual seagrass disturbance. This suggests that excluding the red-edge and near-infrared bands before any IR-MAD analysis may improve results and interpretation if there are significant tidal differences between the bi-temporal image pair.

Spatially coherent patterns by seagrass disturbances were visible in the IR-MAD outputs for the simple 3-band RGB sensor, even though the disturbances were only weakly to moderately separable from other categories. This may provide a more accessible remote sensing option by using consumer-grade UAVs with inbuilt RGB cameras to capture and analyse bi-temporal image pairs for seagrass disturbance. Orthorectified RGB images have been used to map seagrass beds, even radiometrically uncalibrated data^28,58,59^. However, the poorer separability values for the RGB data compared to the 6-band VIS-6 sensor demonstrated here highlight the trade-off between achieving improved change detection results using IR-MAD and relative sensor cost and accessibility.

Physical disturbances, especially root-rhizome damage, can lead to further degradation through positive feedback mechanisms as the sediment is destabilised increasing bed exposure to erosion from waves and currents^60–62^. This may lead to the release of stored soil carbon and negative impacts on future carbon sequestration^63,64^. Additionally, physical disturbances to seagrass beds can impact the composition and condition of faunal communities^65–67^. Management interventions, including educating fishers and boaters to reduce propeller damage, restricting activities under certain tidal conditions, or restoring seagrass beds damaged by watercraft can lead to measurable improvements in seagrass condition^21,68,69^. Timely detection of fine-scale physical disturbances such as propeller scars, which are otherwise difficult to detect, is needed to support the implementation of these intervention strategies. Applying IR-MAD to UAV images provides a relatively simple unsupervised method for identifying disturbance.

Effective long-term monitoring is important for the success of seagrass restoration projects^70,71^ and monitoring cost effectiveness is particularly crucial for projects limited by financial constraints^72^. The effectiveness of blue carbon restoration projects for generating high-quality and verifiable carbon offsetting services requires robust approaches for identifying and tracking change in biomass extent at ecologically relevant spatio-temporal scales. Although IR-MAD cannot provide quantitative measurements of seagrass blue carbon stocks, in detecting fine scale change from baseline conditions, results can complement existing measurement approaches for project reporting.

Change detection using IR-MAD could be further enhanced by adding pre- or post-processing steps. Water column correction, applied to the UAV images, may assist in reducing change signals caused by differences in tidal level between images. Additionally, changes detected in IR-MAD outputs could be automatically extracted using object-based image analysis methods, image thresholding, or machine learning methods. IR-MAD outputs by themselves, however, offer a valuable resource for coastal managers and scientists due to the unsupervised nature of the process, and the need for no data inputs beyond two coregistered UAV images.

Wider application of IR-MAD for seagrass change detection requires further testing over multi-species seagrass beds and different water optical properties. The study findings are restricted to two temperate water study sites covering predominantly monospecific seagrass beds. Additionally, the band configurations (including band centres and widths) of the two virtual sensors used in this study (VIS-6 and RGB) may not correspond with actual, commercially available sensors. Testing the IR-MAD method using images captured from alternative, commercially available sensors such as a simple RGB sensor commonly integrated on many UAV platforms, may provide further insights into sensor selection considerations for coastal managers.

## Conclusions

Seagrasses perform a range of vital ecosystem functions including sediment stabilisation, carbon sequestration, habitat provision and water purification^73^. There is a need for seagrass monitoring techniques that detect disturbances which interfere with these functions. The application of IR-MAD to co-registered, bi-temporal UAV-acquired images offers a relatively cost-effective, unsupervised method of detecting very fine-scale changes to seagrass beds, at the on-demand temporal resolution UAV imaging provides. IR-MAD applied to UAV-acquired multispectral images can be used to distinguish and map multiple forms of change in seagrass beds, including propeller scars, regrowth, and oyster farming. In detecting key fine-scale change features, this method can inform management interventions designed to prevent seagrass degradation, guide restoration efforts, and contribute to blue carbon accounting. Change detection using IR-MAD is unsupervised, can be implemented in open-source software, with consumer-grade computing power, using images from standard off-the-shelf UAV multispectral sensors.

## Data Availability

The datasets generated and analysed during the current study are available from the corresponding author on request.
